# APE1 shRNA‐loaded cancer stem cell‐derived extracellular vesicles reverse Erlotinib resistance in non‐small cell lung cancer via the IL‐6/STAT3 signalling

**DOI:** 10.1002/ctm2.876

**Published:** 2022-05-23

**Authors:** Chun‐Han Tang, Ling Qin, Ying‐Chun Gao, Tai‐Yu Chen, Ke Xu, Tao Liu, Tao Ren

**Affiliations:** ^1^ Clinical Medical College Chengdu Medical College Chengdu P. R. China; ^2^ Department of Gastroenterology Clinical Medical College, The First Affiliated Hospital of Chengdu Medical College Chengdu P. R. China; ^3^ Oncology Department Pengzhou People's Hospital Chengdu P. R. China; ^4^ Oncology Department The First Affiliated Hospital of Chengdu Medical College Chengdu P. R. China

**Keywords:** APE1, cancer stem cells, drug resistance, Erlotinib, extracellular vesicles, interleukin‐6, non‐small cell lung cancer, short hairpin RNA, signal transducers and activators of transcription 3

## Abstract

**Objective:**

Apurinic endonuclease 1 (APE1) has been suggested as an oncogene of lung tumours and our bioinformatics analysis identified the association between Erlotinib resistance and interleukin‐6 (IL‐6). Thus, we performed this work to delineate the mechanistic actions of APE1/IL‐6 signalling in Erlotinib resistance of non‐small cell lung cancer (NSCLC).

**Methods:**

We selected human NSCLC cell lines HCC827 and PC9 to establish Erlotinib‐resistant HCC827R and PC9R cells. Cancer stem cells (CSCs) were isolated from Erlotinib‐sensitive HCC827P and PC9P cells (PCSCs) and from HCC827R and PC9R cells (RCSCs). Further, extracellular vesicles (EVs) were separated from PCSCs (PCSC‐EVs) and RCSCs (RCSC‐EVs) and co‐cultured with RCSCs with or without short hairpin RNA (shRNA)‐targeting APE1 (APE1 shRNA) transduction. In addition, functional assays were conducted to determine the effect of APE1 shRNA on malignant phenotypes of cancer cells in vitro and in vivo and the activation of IL‐6/STAT3 signalling.

**Results:**

It was found that NSCLC cells could internalize both RCSC‐EVs and PCSC‐EVs. RCSC‐EVs augmented the resistance of NSCLC cells to Erlotinib. The overexpression of APE1 occurred in NSCLC tissues, and IL‐6 was enriched in serum samples of patients with NSCLC. APE1 shRNA was demonstrated to restrict the Erlotinib resistance of NSCLC cells by inactivating the IL‐6/STAT3 signalling. Additionally, shAPE1‐loaded RCSC‐EVs suppressed the Erlotinib resistance of NSCLC via the IL‐6/STAT3 axis both in vitro and in vivo, as reflected by impeded malignant phenotypes and xenograft tumour formation.

**Conclusions:**

Collectively, these data indicate that APE1 confers Erlotinib resistance by activating the IL‐6/STAT3 signalling, suggesting targeting APE1 as a possible therapeutic target in Erlotinib‐resistant NSCLC.

## Graphical abstract

Collectively, these data indicate that APE1 confers Erlotinib resistance by activating the IL‐6/STAT3 signaling, suggesting targeting APE1 as a possible therapeutic target in Erlotinib‐resistant NSCLC.

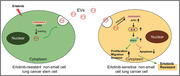

## 1 INTRODUCTION

Lung cancer represents the leading cause of worldwide cancer‐related mortality and is histologically categorized into non‐small cell lung cancer (NSCLC) and small cell lung cancer, with NSCLC accounting for the majority of lung cancer.^1^, ^2^ Advancements have been achieved in the treatment of this cancer over the past two decades, such as targeted therapy and immunotherapy, but treatment resistance restricts the clinical application.^3^, ^4^, ^5^ Understanding and clarifying the biology of the resistance mechanism of NSCLC can thus contribute to more precise therapies and advances in the treatment of NSCLC.

Mutation of epidermal growth factor receptor (EGFR) gene has emerged as a critical issue in the management of lung cancer.^6^ Activating EGFR mutation can be identified in about 15% of lung cancer cases in White populations and in approximately 50% of Asian cases.^7^ Erlotinib, a subtype of small‐molecule EGFR tyrosine kinase inhibitors (TKIs), has been the first‐line treatment in patients suffering from EGFR‐mutant lung cancer in a number of countries.^6^, ^8^, ^9^ However, many patients with the Erlotinib treatment inevitably become resistant to the drug within 9 to 13 months, which weakens the antitumor effect of Erlotinib.^10^ Herein, our work tried to explore the potential role of cancer stem cells (CSCs) in the Erlotinib resistance of NSCLC cells with the aim to discover novel targets for the improvement of Erlotinib efficacy.

Intriguingly, CSC‐derived extracellular vesicles (EVs) may exert tumour‐promoting effects through functioning as paracrine factors.^11^, ^12^ Further to investigate underlying molecular mechanism, we noticed that the up‐regulation of interleukin‐6 (IL‐6) and STAT has been highlighted as a potential driver of EGFR‐TKIs resistance.^13^ A study on the natural compound polyphyllin I pointed out that the regulation of IL‐6/STAT3 signalling EGFR‐TKI may reverse epithelial‐to‐mesenchymal transition and serve as a potential new way to overcome the EGFR‐TKI resistance in lung cancer.^14^ In addition, extracellular human apurinic endonuclease 1 (APE1) can trigger the activation of transcription factor nuclear factor gammaB, promote the IL‐6 signalling, and thereby augment IL‐6‐induced autocrine and paracrine actions.^15^ Taken together, we hypothesized that the APE1/IL‐6/STAT3 signalling might have a role to confer in the drug resistance of NSCLC to Erlotinib, and that the construction of CSC‐derived EVs targeting this signalling may serve as a promising approach to reverse the Erlotinib resistance in NSCLC.

## 2 METHODS

### 2.1 Bioinformatics analysis

An Erlotinib resistance‐related GSE69181 microarray was obtained from the data platform GPL571, which includes array data of four patients. With |log2(FoldChange)| > 1 and *p* < .05 as the criteria, the R “limma” package was adopted to screen differentially expressed genes (DEGs). Lung cancer–related genes were retrieved from the GeneCards database, during which, with “lung cancer” as the keyword, 8301 genes were downloaded and then ranked according to the Relevance score from large to small. Next, the Relevance score ≥10 was set as the threshold and the genes related to lung cancer were screened, with a retrieval time of 2021.01. A Venn diagram of DEGs and lung cancer–related genes was plotted to determine the intersected genes. Protein–protein interaction (PPI) analysis of 33 genes was carried out through the STRING database, with human as the species, and an interaction network was drawn using Cytoscape software (v3.8.2). The GEPIA2 database was used to analyse the expression of APE1 in the tumour tissue samples of lung cancer patients and normal tissue samples. At the same time, APE1 expression and clinical data were combined for survival analysis.

### 2.2 Sample collection

Cancer and adjacent normal tissues were harvested from 67 patients with NSCLC (45 males and 22 females, aged 37–78 years) undergoing surgery at the First Affiliated Hospital, Clinical Medical College of Chengdu Medical College from June 2017 to June 2019. As per the 2015 WHO Classification of Tumours of the Lung, all patients were classified with the TNM staging system—28 cases at stages I–II and 39 cases at stage IIIa. Patients who meet the following requirements were included: (1) patients had no other malignant tumours; (2) patients had not received chemotherapy, radiotherapy and other treatments before the surgery; (3) the NSCLC sample was confirmed by pathologists. Meanwhile, peripheral blood was collected from 35 healthy donors as the control and also from lung cancer patients. The study, approved by the Ethics Committee of the First Affiliated Hospital, Clinical Medical College of Chengdu Medical College (2022CYFYIRB‐BA‐Feb23), with informed consents obtained from all patients, was conducted in compliance with the *Declaration of Helsinki*.

### 2.3 Cell culture and treatment

Human NSCLC cell line HCC827 (CRL‐2868; ATCC, Manassas, VA) was cultured in 1640 medium (21875034, Invitrogen) with 10% FBS (16000‐044, Invitrogen). The doubling time of the HCC827 cell line was 28–60 h. The medium was changed three times a week and cells were subcultured at a 1:3 split ratio each time. Human NSCLC cell line PC9 (CM‐H210, Gaining Biological, Shanghai, China) and human kidney epithelial cell line 293T (CRL‐3216, ATCC) were cultured in 10% FBS‐containing DMEM (C11960500BT, Invitrogen). The doubling time of PC9 cell line was 30–60 h. The medium was changed three times a week and cells were subcultured at a 1:3 split ratio each time. The doubling time of 293T cell line was 24–48 h. The medium was changed four times a week and cells were subcultured at a 1:3 split ratio each time. All cells were incubated under 37°C and 5% CO_2_. Cells that were passaged over three times were used for subsequent experiments.

Erlotinib (E‐4997, LC Laboratories, Woburn MA), at the initial concentration of 10 mM, was dissolved in DMSO (67‐68‐5, Sigma), after which an Erlotinib‐resistant HCC827 and PC9 cell lines (referred to as HCC827R and PC9R, respectively) were developed as previously described.^16^, ^17^ Briefly, parental HCC827 (HCC827P) and PC9 (PC9P) cells were first cultured in .5‐μM Erlotinib‐containing medium and then in 1‐, 1.5‐, 2‐, 2.5‐, 3‐, 3.5‐, 4‐, 4.5‐ and 5‐μM Erlotinib‐containing medium. After a 5‐month passage, cells that can grow in a 5‐μM Erlotinib‐containing medium were resistant cells. The doubling time of resistant cells was 48–72 h. The medium was changed three times a week, and cells were subcultured at a 1:3 split ratio each time. Cells were incubated with 5% CO_2_ at 37°C. Cells passaged over 60 times were selected for subsequent experiments. All cells were tested for mycoplasma monthly to prevent cell contamination and cross‐contamination.

### 2.4 Isolation and identification of CSCs

HCC827P, PC9P, HCC827R and PC9R cells were subjected to flow cytometric analysis to obtain CD133^+^ABCG2^+^ CSCs (labelled HCC827P‐CSCs, PC9P‐CSCs, HCC827R‐CSCs and PC9R‐CSCs, respectively), with corresponding antibodies against CSC markers (anti‐CD133, ab216323, Abcam, Cambridge, UK; anti‐ABCG2, ab229193 Abcam) used in the analysis.

CSCs were cultured in serum‐free DMEM with 50‐mg/ml insulin (A1895602, Gibco), 100‐mg/ml transferrin (T2872, Gibco), 10‐mg/ml tetramethylenediamine (T21407, Sigma), .03‐mM sodium selenite (10102‐18‐8, Sigma), 2‐mM progesterone (15775‐74‐3, Sigma), .6% glucose (A2494001, Gibco), 5‐mM HEPES (15630080, Gibco), .1% sodium bicarbonate (144‐55‐8, Sigma), .4% bovine serum albumin (30063788, Gibco), 2‐mM l‐glutamine (G8540‐25G, Sigma), 50‐IU/ml penicillin (36945‐98‐9, Sigma) and 50‐mg/ml streptomycin (3810‐74‐0, Sigma), 20‐mg/ml EGF (PHG0313, Life Technologies, Foster City, CA) and 10‐mg/ml bFGF (PHG0261, Life Technologies) with 100% humidity and 5% CO_2_ at 37°C. After the incubation, CSCs were trypsinized and harvested.

Detection of CD166^+^ (CD166‐PE, 559263, BD Biosciences, San Jose, CA) and CD44^+^ (CD44‐FITC, 555478, BD Biosciences) using flow cytometry and in vitro sphere‐forming assay were conducted for the identification of CSCs.

### 2.5 Sphere‐forming assay

The isolated and purified CSCs were cultured in a serum‐free medium containing EGF (20 ng/ml, PHG0313, Life Technologies, Foster City, CA), bFGF (10‐ng/ml, PHG0261, Life Technologies) and B27 additive (A1895602, Life Technologies). The sphere‐forming ability of cells was observed on the 2nd and 7th day, respectively.

### 2.6 Lentivirus‐mediated transduction

Using Lipofectamine 3000 reagent (L3000001, Invitrogen), 293T cells were transfected with pLenti‐shAPE1 (plasmids carrying short hairpin RNA [shRNA] targeting APE1) or the corresponding NC pLenti‐sh‐NC, pCMV‐VSV‐G and pCMV‐Delta8.9, respectively. Different sequences of shAPE1 were as follows: shAPE1–1: 5′‐GCCTGGACTCTCTCATCAATA‐3′; shAPE1–2: 5′‐GCAGTGATCACTGTCCTATCA‐3′ and shAPE1–3: 5′‐GCAAACCTGCCACACTCAAGA‐3′, among which the sequence that presented the optimal silencing efficiency was selected for subsequent experiments.

Transfected cells were transferred to 5% FBS‐containing medium (12 ml) 20 h later for another 48‐h incubation. The lentivirus‐containing supernatant was then harvested, filtered and then stored at −80°C. For the construction of APE1‐silencing HCC827P‐CSCs, PC9P‐CSCs, HCC827R‐CSCs and PC9R‐CSCs, cells of 50% confluence were incubated in a lentivirus‐containing medium for 8 h and subsequently in DMEM containing 10% FBS for 24 h, with 10‐μg/ml puromycin (540411, Sigma) added to screen out stably transduced cells.

### 2.7 Isolation of EVs

Following 48–72 h of CSC culture in a medium supplemented with EV‐free phosphate‐buffered saline (PBS), the culture medium (CM) of CSCs was collected and subjected to ultracentrifugation to extract EVs. Specifically, collected CM was centrifuged successively (500 *g* for 15 min; 2000 *g* for 15 min; 10 000 *g* for 20 min), followed by filtering using a .22‐μm filter and another 70‐min centrifugation at 110 000 *g*. The EV extract was then resuspended in PBS and centrifuged again in the same way, the extract of which was again resuspended in 100‐μl‐sterilized PBS to obtain EVs secreted by HCC827R‐CSCs/PC9R‐CSCs (HCC827R‐CSC‐EVs/PC9R‐CSC‐EVs, collectively known as RCSC‐EVs) and by HCC827P‐CSCs/PC9P‐CSCs (HCC827P‐CSC‐EVs/PC9P‐CSC‐EVs, collectively known as PCSC‐EVs).

### 2.8 EV characterization

The particle size of EVs was determined via NTA using Malvern's NanoSight NS300. EVs were resuspended in 1‐ml PBS and mixed with filtered PBS serving as a control. Diluted EVs were injected into NanoSight LM10 instrument to measure their particle sizes. The conditions for NTA were 23.75 ± .5°C, and the measurement time was 60 s.

EV morphology was observed through the Hitachi H‐7650 transmission electron microscope (TEM). Briefly, EV samples (10 μl) were loaded on copper grids for 20 min. The grids were washed twice with PBS and dried with filter paper. Images were observed under a TEM at 100 keV.

Further, EVs were lysed with a radioimmunoprecipitation assay buffer, followed by protein quantification via a Bicinchoninic Acid Kit (23225, Thermo Fisher Scientific, Rockford, IL). Western blot was adopted to determine the expression of EV surface positive protein markers CD63 (ab216130, 1:1000) and TSG101 (ab30871, 1:1000) as well as that of endoplasmic reticulum protein marker Calnexin (ab22595, 1:1000, serving as NC).

### 2.9 EV uptake by NSCLC cells

EVs were labelled with PKH67 green fluorescent (PKH67GL, Sigma).^18^ In brief, EVs were suspended in 1‐ml Diluent C reagent and added with 4 μl of PKH67 dye. Next, PKH67‐labelled EVs were co‐cultured for 12 h with HCC827P and PC9P cells at 37°C. Cells were incubated with DAPI (.5‐μg/ml; D1306, Invitrogen) to stain nuclei, followed by a fluorescence microscopic observation of PKH67. To detect the transfer of mRNA from EVs to HCC827P and PC9P cells, RCSC‐EVs or PCSC‐EVs, cells were co‐cultured with the EVs for 12 h and subjected to qRT‐PCR detection of mRNA expression, with primer sequences depicted in Table S1.

### 2.10 CCK‐8 assay

Cell viability was observed based on the protocols of a CCK‐8 kit (96992, Sigma). Briefly, cells in 96‐well plates (5 × 10^3^ cells/well) were incubated with Erlotinib at different concentrations or 10‐μg EVs for 48 h. After that, cells were incubated with 10 μl of a freshly prepared CCK‐8 reagent at 37°C for 1 h, followed by the detection of OD at 450 nm. The concentration of Erlotinib, which caused 50% inhibition (IC50) cells, was calculated.

### 2.11 Flow cytometry

After 48 h of cell transduction, cells were trypsinized, placed in flow tubes and centrifuged. The cell pellet was centrifuged again. An Annexin V‐FITC/PI staining reagent was prepared following the protocols of the Annexin‐V‐FITC apoptosis kit (K201‐100, BioVision, USA). Afterwards, FITC and PI fluorescence was detected, respectively, using 525‐ and 620‐nm band‐pass filters at an excitation wavelength of 488 nm.

### 2.12 Transwell assay

Transwell invasion assay was conducted with 6.5‐mm Transwell chambers (8‐μM pores). Briefly, Matrigel was incubated in the upper Transwell chamber for 2–3 h to obtain a Matrigel‐coated system. Afterwards, cells (1 × 10^5^) were suspended and supplemented to the upper chamber (200 μl/well), while 800 μl of 10% FBS‐containing CM to the lower chamber, followed by 20–24 h of incubation. The plate was stained with .5% crystal violet, followed by the observation of invasive cells utilizing an inverted microscope.

Transwell migration assay was conducted in the same way, except that the Matrigel was not included and the incubation period was 16 h. Cell counting was carried out in at least randomly selected four fields in microscopic observation.

### 2.13 ELISA

IL‐6 level was measured with the IL‐6 ELISA kit (D6050, R&D Systems, Minneapolis, MN). Briefly, 1 × 10^3^ cells, 50 μl of serum after centrifugation or 50 μl of supernatant collected after tissue lysis were plated into a 96‐well plate and incubated. The OD450 was determined using a microplate reader.

### 2.14 RNA quantification

Total RNA from cells or tissues was extracted utilizing a TRIzol reagent (10296010, Invitrogen), 1 μg of which was applied for cDNA synthesis. qRT‐PCR was followed using SYBR Premix ExTaqTMII and ABI PRISM 7900HT System. Using β‐actin as the housekeeper gene, the relative mRNA expression of tested gene was calculated with 2^−△△CT^. Primers involved in our work are listed in Table S1.

### 2.15 Western blot

Total protein was extracted, separated and transferred to membranes, which were then blocked with 5% skim milk. Next, the protein‐loaded membrane was probed with antibodies against APE1 (ab92744, 1:500, Abcam), MDR1 (ab170904, 1:500), MRP (ab261871, 1:500), LRP (ab273093, 1:500), ABCG2 (ab207732, 1:500), STAT3 (ab68153, 1:500, Abcam), p‐STAT3 (ab267373, 1:1000, Abcam), Bcl‐2 (ab196495, 1:1000, Abcam), Bax (ab53154, 1:500, Abcam), cleaved caspase‐3 (ab2302, 1:1000, Abcam) and β‐actin (ab8227, 1:500, Abcam) and then with HRP‐labelled anti‐rabbit IgG secondary antibodies (No. 7074, 1:5000; CST, Danvers, MA) at 37°C for 1 h. The blots were visualized via an ECL reagent (32106, Thermo Fisher Scientific), normalized to β‐actin.

### 2.16 Animal experiments

Forty SPF female BALB/C nude mice (15–18 g, 6‐week old; Hunan SJA Laboratory Animal, Changsha, Hunan, China) were housed individually in the SPF laboratory (22–25°C, 60%–65% humidity, a 12‐h light/dark cycle). They were free to access food and water for 1‐week acclimation prior to the experiment, with health observed before the experiment.

The mice were randomly allotted into five groups (*n* = 8): Control group (mice injected only with HCC827P cells), Erlotinib group (mice injected with 10‐μM Erlotinib alone), Erlotinib + HCC827R‐CSC‐EVs group (mice injected with 10‐μM Erlotinib + 100‐μg HCC827R‐CSC‐EVs), Erlotinib + HCC827R‐CSC‐EVs shNC groups (mice injected with 10‐μM Erlotinib + 100‐μg HCC827R‐CSC‐EVs shNC), Erlotinib + HCC827R‐CSC‐EVs shAPE1 (mice injected with 10‐μM Erlotinib + 100‐μg HCC827R‐CSC‐EVs shAPE1).

Specifically, HCC827P cells (5 × 10^6^ cells/ml) were injected subcutaneously into the armpit of mice; upon the tumour size reaching 100–150 mm^3^, HCC827R‐CSC‐EVs or Erlotinib was injected subcutaneously into the tumour xenograft of mice on the 5th, 10th, 15th, 20th and 25th day, respectively.^19^ On the 28th day, mice were euthanized. The volume and weight of xenografts were analysed. These experiments, approved by the Animal Ethics Committee of the First Affiliated Hospital, Clinical Medical College of Chengdu Medical College (2021‐001), were in‐line with *Guide for the Care and Use of Laboratory animals* (NIH).

### 2.17 Statistical analysis

Statistical analysis for data included unpaired *t*‐test for data comparison between two groups, one‐way ANOVA with Tukey's post‐hoc test for data comparison between multiple groups or Bonferroni‐corrected repeated measures ANOVA for data comparison between multiple groups at different time points. The Kaplan–Meier method with a log‐rank test was adopted to assess survival. Measurement data were expressed as mean ± SD. *p* < .05 suggests statistical difference.

## 3 RESULTS

### 3.1 NSCLC cells can internalize CSC‐EVs

**FIGURE 1:**
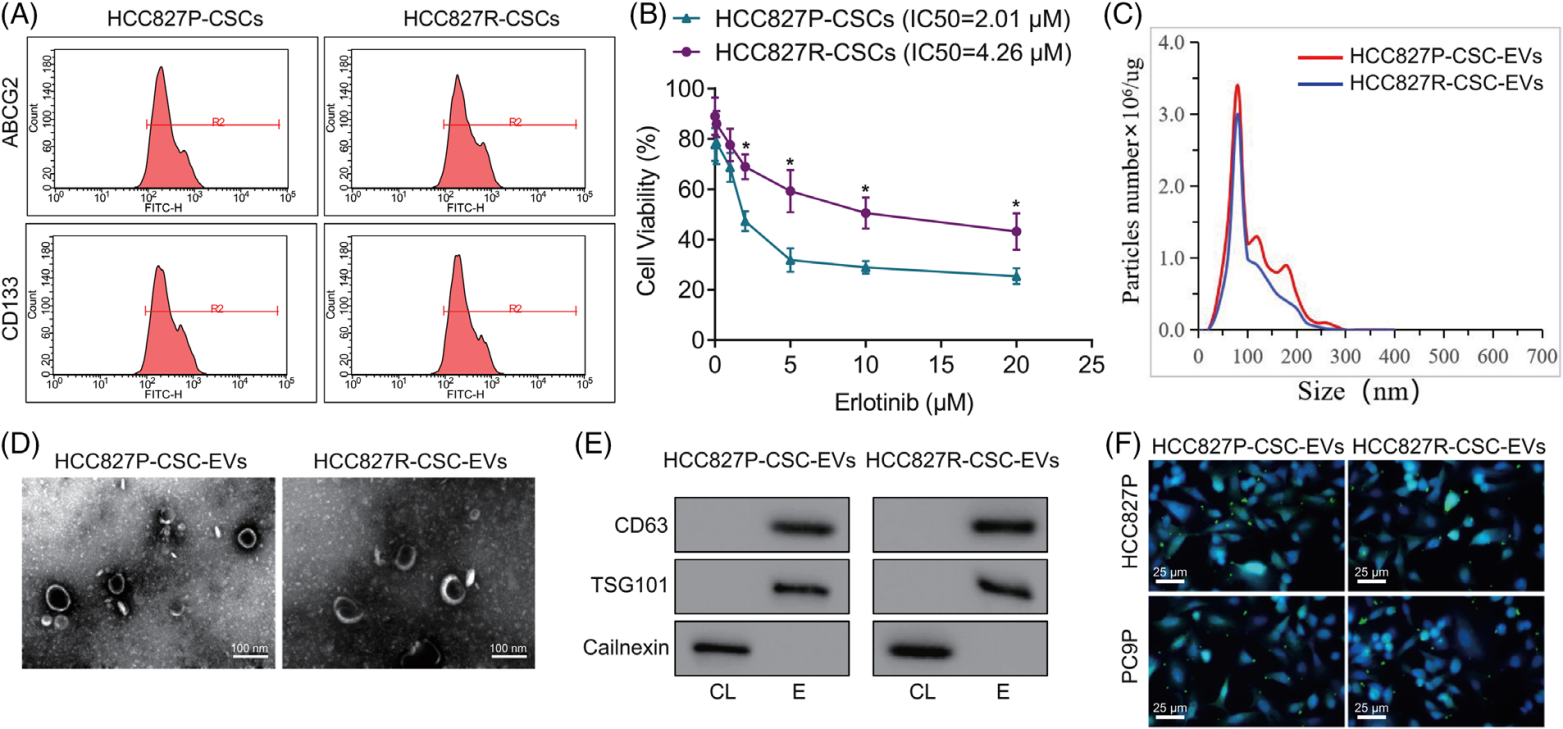
Isolation and characterization of CSC‐EVs as well as their internalization by NSCLC cells. (A) Expression of CSC surface markers (ABCG2 and CD133) in HCC827P‐CSCs and HCC827R‐CSCs, detected by flow cytometry. (B) Cell viability of HCC827P‐CSCs and HCC827R‐CSCs treated with different concentrations of Erlotinib, detected by CCK‐8 assay. (C) Size distribution of EVs derived from HCC827P‐CSCs and HCC827R‐CSCs measured by NTA. (D) Images of TEM observation of HCC827P‐CSCs and HCC827R‐CSCs. (E) Protein bands of EV surface markers (CD63, TSG101 and Calnexin) in Western blot of EVs (labelled E) and cell lysate (labelled CL) in HCC827P‐CSC‐EVs and HCC827R‐CSC‐EVs. (F) Images photographed in fluorescence microscopic observation of green fluorescence PKH67 in HCC827P and PC9P cells following co‐culture with PKH67‐labelled HCC827P‐CSC‐EVs and HCC827R‐CSC‐EVs (DAPI‐labelled nuclei are in blue). * *p* < .05, compared to HCC827P‐CSCs. Each cell experiment was conducted in triplicate. CSC, cancer stem cell; EV, extracellular vesicle; NSCLC, non‐small cell lung cancer; TEM, transmission electron microscope

To investigate the effect of CSCs on the Erlotinib resistance of NSCLC, we isolated lung CSCs from Erlotinib‐sensitive HCC827P cells and HCC827R cells, respectively. Flow cytometry results showed that both HCC827P‐CSCs and HCC827R‐CSCs expressed CSC surface protein markers ABCG2 and CD133 (Figure 1A). In addition, it has been reported that lung CSCs often express CD44 and CD166.^20^ Subsequently, the expression of CD44 and CD166 was further identified by flow cytometry. The results indicated that about 90% was CD44^+^CD166^+^ double positive cells in the isolated CD133^+^ABCG2^+^ HCC827P‐CSCs and HCC827R‐CSCs (Figure S1A). In addition, the results of the sphere‐forming experiment also exhibited that the isolated HCC827P‐CSCs and HCC827R‐CSCs had excellent sphere‐forming ability (Figure S1B). The previous results indicated the successful isolation of HCC827P‐CSCs and HCC827R‐CSCs.

We then exposed HCC827P‐CSCs and HCC827R‐CSCs to increasing concentrations of Erlotinib. HCC827R‐CSCs were found to be more resistant to Erlotinib and had an obviously higher IC50 value of Erlotinib relative to HCC827P‐CSCs (*p* = .009) (Figure 1B).

Further, EVs were isolated from HCC827P‐CSCs and HCC827R‐CSCs via ultracentrifugation, which presented with a diameter distribution from 30 to 200 nm (Figure 1C) and were in a cupped or spherical shape (Figure 1D). Meanwhile, the isolated EVs exhibited up‐regulated expression of CD63 and TSG101 as well as extremely down‐regulated expression of Calnexin, which can be detected only in cell lysates (Figure 1E). These results indicated that EVs were successfully extracted and purified.

Then, the uptake of HCC827R‐CSC‐EVs and HCC827P‐CSC‐EVs by HCC827P and PC9P cells was tested. Green fluorescence dye PKH67 was used to label HCC827R‐CSC‐EVs and HCC827P‐CSC‐EVs, which were then co‐cultured with HCC827P and PC9P for 3 h. Under a fluorescence microscope, a large amount of green fluorescence was observed in the cytoplasm of HCC827P and PC9P cells (Figure 1F), showing the existence of a large number of EVs in HCC827P and PC9P cells. Collectively, these results demonstrated that CSC‐EVs were successfully isolated and that CSC‐EVs could be internalized by NSCLC cells.

### 3.2 RCSC‐EVs promote the resistance of NSCLC cells to Erlotinib

**FIGURE 2:**
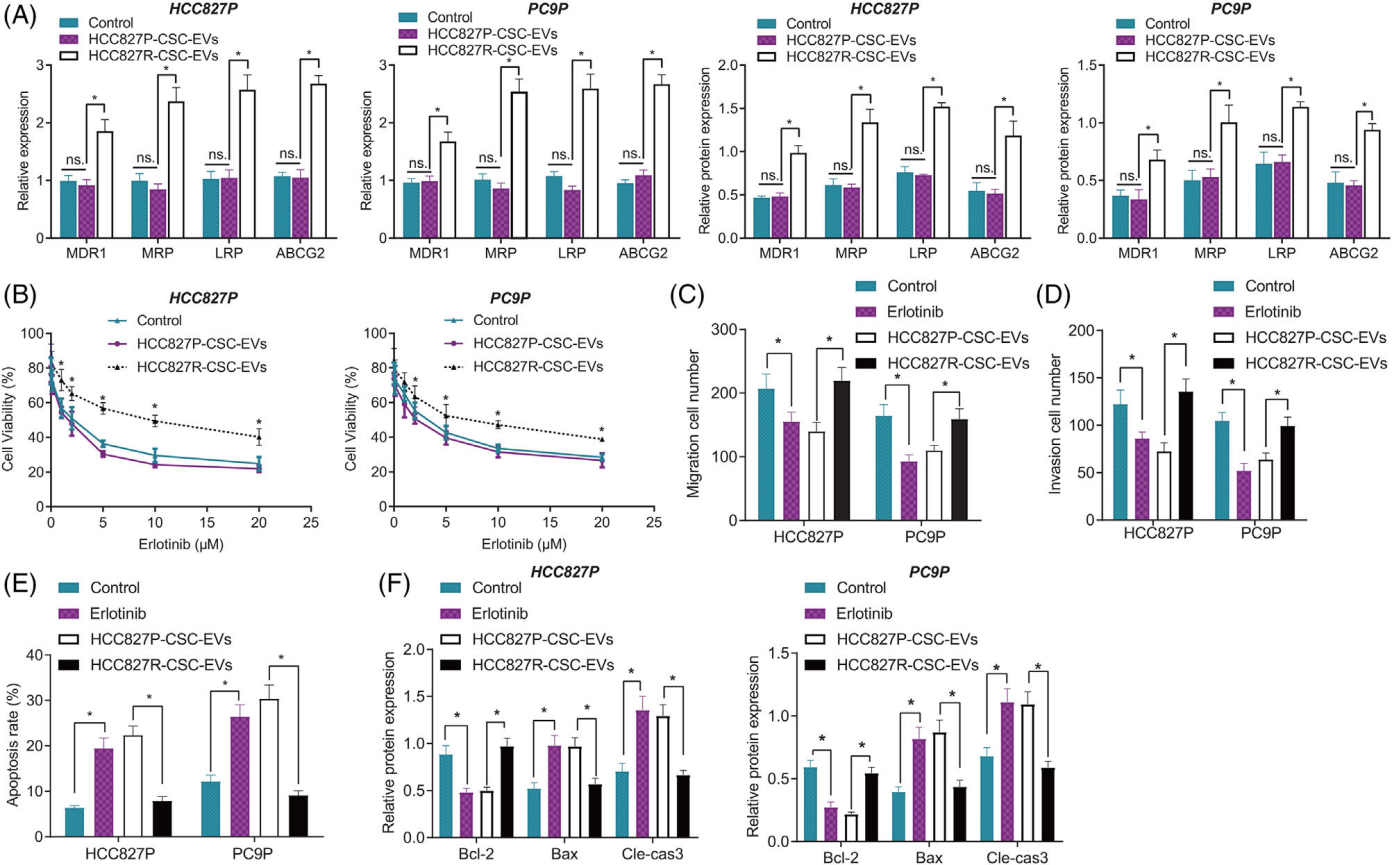
Effect of HCC827P‐CSC‐EVs and HCC827R‐CSC‐EVs on the resistance of NSCLC cells to Erlotinib. (A) Expression of Erlotinib resistance‐related genes MDR1, MRP, LRP and ABCG2 in HCC827P and PC9P cells following co‐culture with HCC827P‐CSC‐EVs or HCC827R‐CSC‐EVs, detected by qRT‐PCR and Western blot. (B) Viability of HCC827P and PC9P cells co‐cultured with HCC827P‐CSC‐EVs or HCC827R‐CSC‐EVs and further treated with different concentrations of Erlotinib, detected by CCK‐8 assay. Quantification of the migration (C) and invasion (D) of Erlotinib (5 μM)‐treated HCC827P and PC9P cells in response to co‐culture with HCC827P‐CSC‐EVs or HCC827R‐CSC‐EVs, observed by Transwell assay. (E) Apoptosis of Erlotinib (5 μM)‐treated HCC827P and PC9P cells in response to co‐culture with HCC827P‐CSC‐EVs or HCC827R‐CSC‐EVs, detected by flow cytometry. (F) Protein expression of anti‐apoptotic Bcl‐2 and pro‐apoptotic Bax and cleaved caspase‐3 in Western blot of Erlotinib (5 μM)‐treated HCC827P and PC9P cells in response to co‐culture with HCC827P‐CSC‐EVs or HCC827R‐CSC‐EVs. * *p* < .05. Each cell experiment was conducted in triplicate. CSC, cancer stem cell; EV, extracellular vesicle; NSCLC, non‐small cell lung cancer

EVs were isolated from HCC827P, PC9P (PCSC‐EVs), HCC827R and PC9R cells (RCSC‐EVs), and we went on to explore the effect of PCSC‐EVs and RCSC‐EVs on the Erlotinib resistance in HCC827P and PC9P cells. qRT‐PCR and Western blot results illustrated no significant alteration in the expression of drug resistance–related genes (MDR1, MRP, LRP and ABCG2) in HCC827P and PC9P cells following co‐culture with PCSC‐EVs, whereas HCC827P and PC9P cells presented with elevated expression of MDR1, MRP, LRP and ABCG2 after co‐culture with RCSC‐EVs (*p* < .001) (Figures 2A and S2A).

Further in the presence of the Erlotinib treatment, HCC827P and PC9P cells co‐cultured with RCSC‐EVs showed much higher cell viability versus those co‐cultured with PCSC‐EVs (Figures 2B and S2B). According to results of the Transwell assay, migratory and invasive potential was reduced (approximately 50%; *p*_HCC827P‐migration_ = .005; *p*_HCC827P‐invasion_ = .002; *p*_PC9P‐migration_ < .001; *p*_PC9P‐invasion_ < .001) in HCC827P and PC9P cells in response to the Erlotinib treatment than those in control cells, but this reduction was reversed by co‐culture with RCSC‐EVs (approximately 50%; *p*_HCC827P‐migration_ < .001; *p*_HCC827P‐invasion_ < .001; *p*_PC9P‐migration_ = .008; *p*_PC9P‐invasion_ = .002) (Figures 2C,D, S2C,D, S3A–B).

Flow cytometric data suggested an increase in the apoptosis of HCC827P and PC9P cells in response to Erlotinib treatment than that in control cells (approximately 50%; *p*_HCC827P_ < .001; *p*_PC9P_ = .001) while a decline was noted following co‐culture with RCSC‐EVs (approximately 70%; *p*_HCC827P_ < .001; *p*_PC9P_ < .001) (Figures 2E and S2E). Western blot results further revealed decreased expression of Bcl‐2 (*p*_HCC827P‐Bcl‐2_ = .002; *p*_PC9P‐Bcl‐2_ = .001), yet increased Bax (*p*_HCC827P‐Bax_ = .003; *p*_PC9P‐Bax_ = .002) and cleaved caspase‐3 (*p*_HCC827P‐cleaved‐caspase‐3_ = .003; *p*_PC9P‐cleaved‐caspase‐3_ = .004) in Erlotinib‐treated HCC827P and PC9P cells, which could be reversed (*p*_HCC827P‐Bcl‐2_ < .001; *p*_PC9P‐Bcl‐2_ < .001; *p*_HCC827P‐Bax_ = .003; *p*_PC9P‐Bax_ = .002; *p*_HCC827P‐cleaved‐caspase‐3_ = .001; *p*_PC9P‐cleaved‐caspase‐3_ = .001) in response to RCSC‐EVs (Figures 2F andS2F).

Taken together, our results indicated that RCSC‐EVs can promote the resistance of NSCLC cells to Erlotinib.

### 3.3 APE1/IL‐6/STAT3 signalling participates in the Erlotinib resistance of NSCLC

**FIGURE 3:**
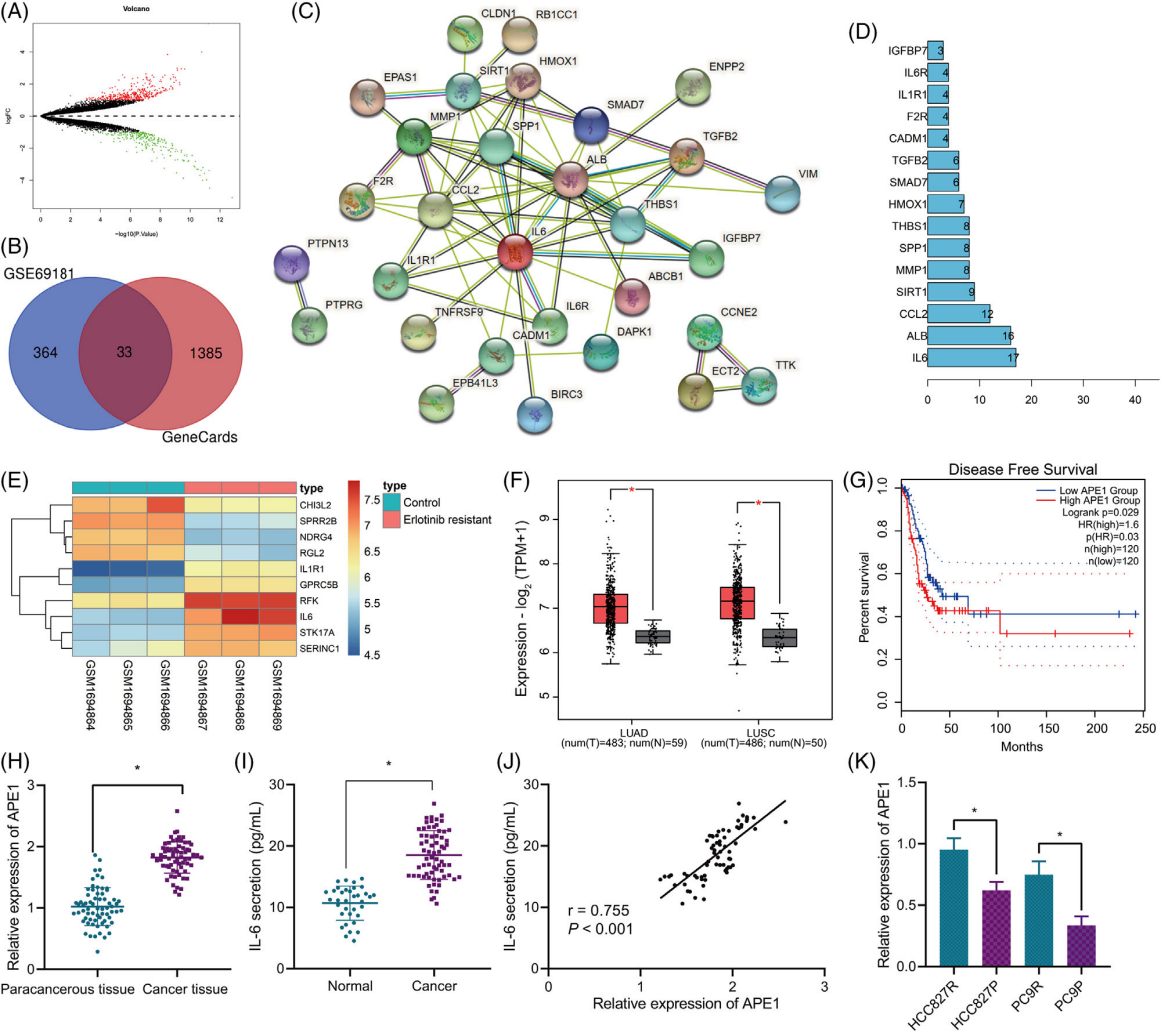
Bioinformatics analysis predicts pathways related to the Erlotinib resistance of NSCLC cells. (A) A volcano plot of Erlotinib resistance–related DEGs in the lung cancer–related GSE69181 microarray. (B) A Venn diagram of up‐regulated DEGs related to Erlotinib resistance and lung cancer–related genes obtained from the GeneCards database, through which 33 genes were identified. (C) PPI analysis of the 33 candidate genes using the String database. (D) Top 15 core genes in the protein interaction network in PPI analysis. (E) A heat map of the expression of IL‐6 in Erlotinib‐resistant samples in the GSE69181 microarray. (F) The expression of APE1 in the tumour and normal tissue samples from the GEPIA2 database. (G) The correlation of APE1 expression with disease‐free survival of lung cancer patients analysed by the GEPIA2 database. The lung cancer patients were divided into a high and a low expression group according to the median value of APE1 expression. (H) The expression of IL‐6 in Erlotinib‐resistant samples in the GSE69181 microarray. qRT‐PCR measurement of APE1 mRNA expression in clinically collected NSCLC tissues and adjacent normal tissues (*n* = 67). (I) ELISA analysis of IL‐6 protein content in serum samples of healthy controls (*n* = 35) and NSCLC patients (*n* = 67). (J) Pearson correlation analysis of APE1 expression and IL‐6 expression in NSCLC tissues (*n* = 67). (K) qRT‐PCR measurement of APE1 mRNA expression in HCC827P, PC9P, HCC827R and PC9R cells. * *p *< .05. Each cell experiment was conducted in triplicate. APE1, apurinic endonuclease 1; DEG, differentially expressed gene; IL‐6, interleukin‐6; NSCLC, non‐small cell lung cancer; PPI, protein–protein interaction

Next, we sought to determine the key signalling involved in the Erlotinib resistance in NSCLC. Analysis of the DEGs in the GSE69181 dataset yielded 397 significantly highly expressed genes associated with the Erlotinib resistance (Figure 3A). Then, 1418 lung cancer‐related genes were obtained from the GeneCards database, and following intersection with the previous 397 genes, 33 candidate genes were identified (Figure 3B). The 33 genes were then subjected to PPI analysis (Figure 3C), based on which IL‐6 was found to be in the core position, and the degree of association was the highest (Figure 3D). Analysis of the DEGs in the Erlotinib‐resistant samples in the GSE69181 dataset revealed that IL‐6 expression was augmented in the Erlotinib‐resistant samples relative to control samples (Figure 3E).

Intriguingly, it has been suggested that the up‐regulation of IL‐6 signal transduction and STAT may be a driver of EGFR‐TKIs resistance,^13^ and APE1 has been established to promote the IL‐6 signalling and thereby augment IL‐6‐induced autocrine and paracrine actions.^15^, ^21^ Collectively, we speculated that the IL‐6/STAT3 signalling may be involved in the regulation of the Erlotinib resistance of NSCLC. Analysis of the GEPIA2 database showed that versus the normal samples, the expression of APE1 was increased in both lung adenocarcinoma (LUAD) and lung squamous cell carcinoma (LUSC) samples (Figure 3F). In addition, APE1 expression was significantly correlated with disease‐free survival in patients with LUAD and LUSC and patients with high APE1 expression had poorer prognosis (Figure 3G).

To examine the speculation, we determined the expression of APE1 and IL‐6 and demonstrated the up‐regulated expression of APE1 (*p* < .001) in NSCLC tissues as well as the up‐regulated IL‐6 level (*p* < .001) in the serum of patients with NSCLC, relative to adjacent normal tissues (Figure 3H,I). In addition, the results of Pearson correlation analysis identified a positive correlation of the expression of APE1 in cancer tissues of NSCLC patients with that of IL‐6 in serum (Figure 3J).

qRT‐PCR results showed higher expression of APE1 in the HCC827R and PC9R cells than that in HCC827P and PC9P cells (*p*_HCC827R vs. HCC827P_ = .008; *p*_PC9R vs. PC9P_ = .002) (Figure 3K).

Thus, the APE1/IL‐6/STAT3 may be the key signalling in the modulation of the Erlotinib resistance of NSCLC.

### 3.4 APE1 enhances the Erlotinib resistance of NSCLC cells via IL‐6/STAT3 signalling activation

**FIGURE 4:**
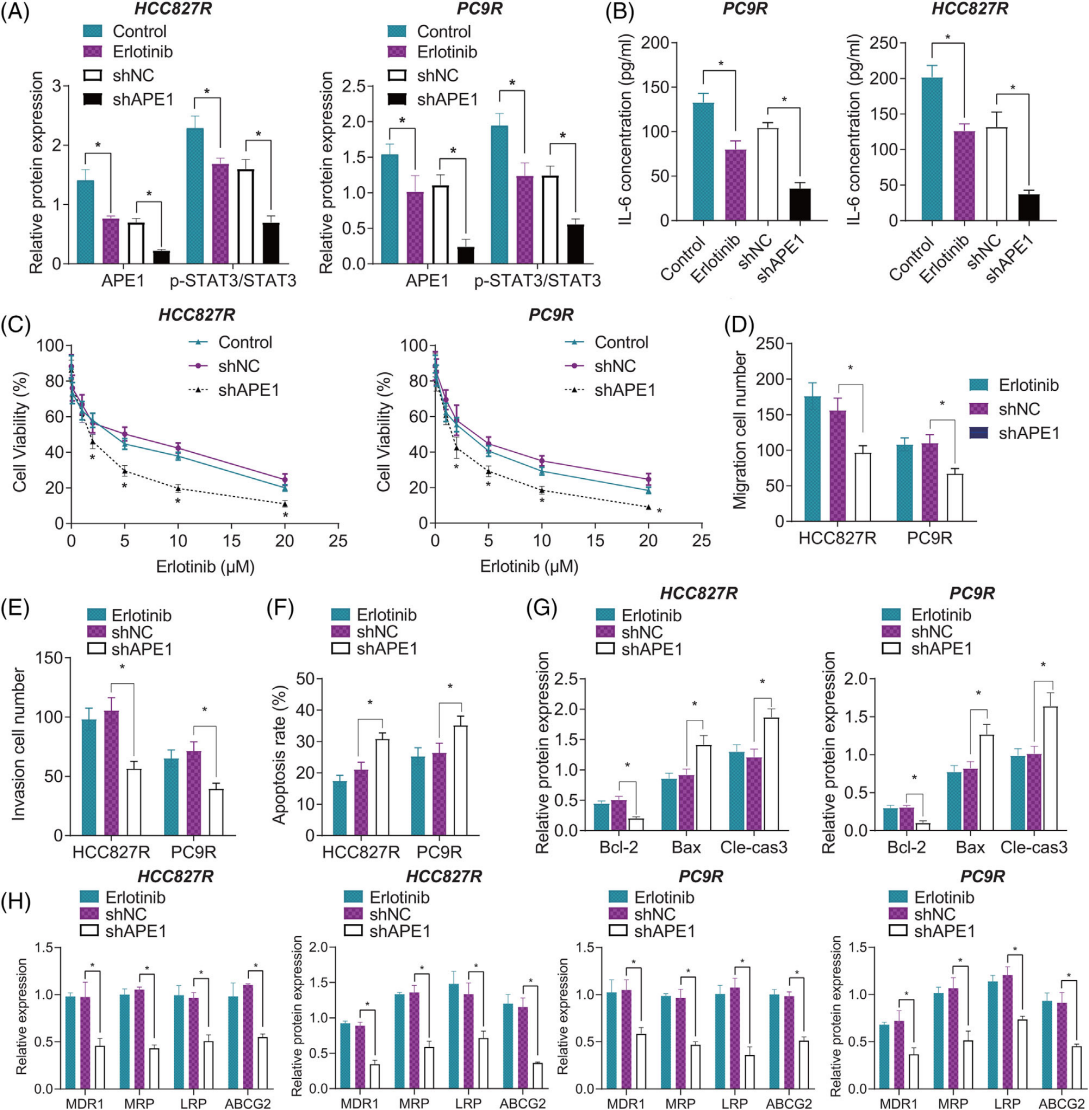
APE1 affects the Erlotinib resistance of NSCLC cells through mediating the IL‐6/STAT3 signalling. (A) The protein expression of APE1 and p‐STAT3/STAT3 in HCC827R and PC9R cells treated by Erlotinib alone or in combination with shAPE1, measured by the Western blot. (B) The content of IL‐6 in the supernatant of HCC827R and PC9R cells treated by Erlotinib alone or in combination with shAPE1, detected by ELISA. (C) Cell viability in HCC827R and PC9R cells treated by Erlotinib alone or in combination with shAPE1, detected by CCK‐8 assay. Quantification of the migration (D) and invasion (E) in HCC827R and PC9R cells treated by Erlotinib alone or in combination with shAPE1, observed in the Transwell assay. (F) The apoptosis of HCC827R and PC9R cells treated by Erlotinib alone or in combination with shAPE1, tested by flow cytometry. (G) The protein expression of Bcl‐2, Bax and cleaved caspase‐3 in HCC827R and PC9R cells treated by Erlotinib alone or in combination with shAPE1, measured by the Western blot. (H) The mRNA and protein expression of MDR1, MRP, LRP and ABCG2 in CC827R and PC9R cells treated by Erlotinib alone or in combination with shAPE1, measured by qRT‐PCR and Western blot. *p < .05. Each cell experiment was conducted in triplicate. APE1, apurinic endonuclease 1; IL‐6, interleukin‐6; NSCLC, non‐small cell lung cancer

Following the aforementioned findings, we then managed to verify the role of the APE1‐mediated IL‐6/STAT3 signalling in the Erlotinib resistance of NSCLC cells. After treatment with shAPE1, the Erlotinib‐induced decrease (*p*_HCC827R‐APE1_ = .004; *p*_HCC827R‐p‐STAT3/STAT3_ = .010; *p*_HCC827R‐IL‐6_ = .001; *p*_PC9R‐APE1_ = .028; *p*_PC9R‐p‐STAT3/STAT3_ = .008; *p*_PC9R‐IL‐6_ < .001) in the expression of APE1 and p‐STAT3/STAT3 in HCC827R and PC9R cells as well as in that of IL‐6 in the cell supernatant was further decreased (*p*_HCC827R‐APE1_ < .001; *p*_HCC827R‐p‐STAT3/STAT3_ = .001; *p*_HCC827R‐IL‐6_ < .001; *p*_PC9R‐APE1_ = .001; *p*_PC9R‐p‐STAT3/STAT3_ = .001; *p*_PC9R‐IL‐6_ < .001) (Figure 4A,B).

Upon different concentrations of Erlotinib, the viability of shAPE1‐treated HCC827R and PC9R cells was always higher than that of shNC‐treated HCC827R and PC9R cells (*p*_HCC827R_ = .003; *p*_PC9R_ = .001) (Figure 4C). Moreover, the migratory (*p*_HCC827R_ < .001; *p*_PC9R_ = .004) and invasive (*p*_HCC827R_ < .001; *p*_PC9R_ = .001) potential of HCC827R and PC9R cells, following treatment with 5‐μM Erlotinib, was shown to be attenuated (approximately 50%) in response to shAPE1 versus shNC (Figures 4D,E and S3C,D). In addition, the 5‐μM Erlotinib augmented the apoptosis of shAPE1‐treated HCC827R and PC9R cells (approximately 30%; *p*_HCC827R_ = .001; *p*_PC9R_ = .002) compared to shNC (Figure 4F). Western blot results indicated that 5‐μM Erlotinib decreased the level of Bcl‐2 (*p*_HCC827R‐Bcl‐2_ = .005; *p*_PC9R‐Bcl‐2_ = .043) and elevated that of Bax (*p*_HCC827R‐Bax_ < .001; *p*_PC9R‐Bax_ < .001) and cleaved caspase‐3 (*p*_HCC827R‐cleaved‐caspase‐3_ < .001; *p*_PC9R‐cleaved‐caspase‐3_ < .001) in HCC827R and PC9R cells in response to shAPE1 versus shNC (Figure 4G).

Moreover, treatment with 5‐μM Erlotinib reduced the expression of MDR1, MRP, LRP and ABCG2 in sh‐APE1‐treated HCC827R and PC9R cells (all *p* < .01) (Figure 4H).

These results collectively suggested that APE1 promoted the resistance of NSCLC cells to Erlotinib through the activation of the IL‐6/STAT3 signalling.

### 3.5 shAPE1‐loaded RCSC‐EVs attenuate the resistance of NSCLC cells to Erlotinib

**FIGURE 5:**
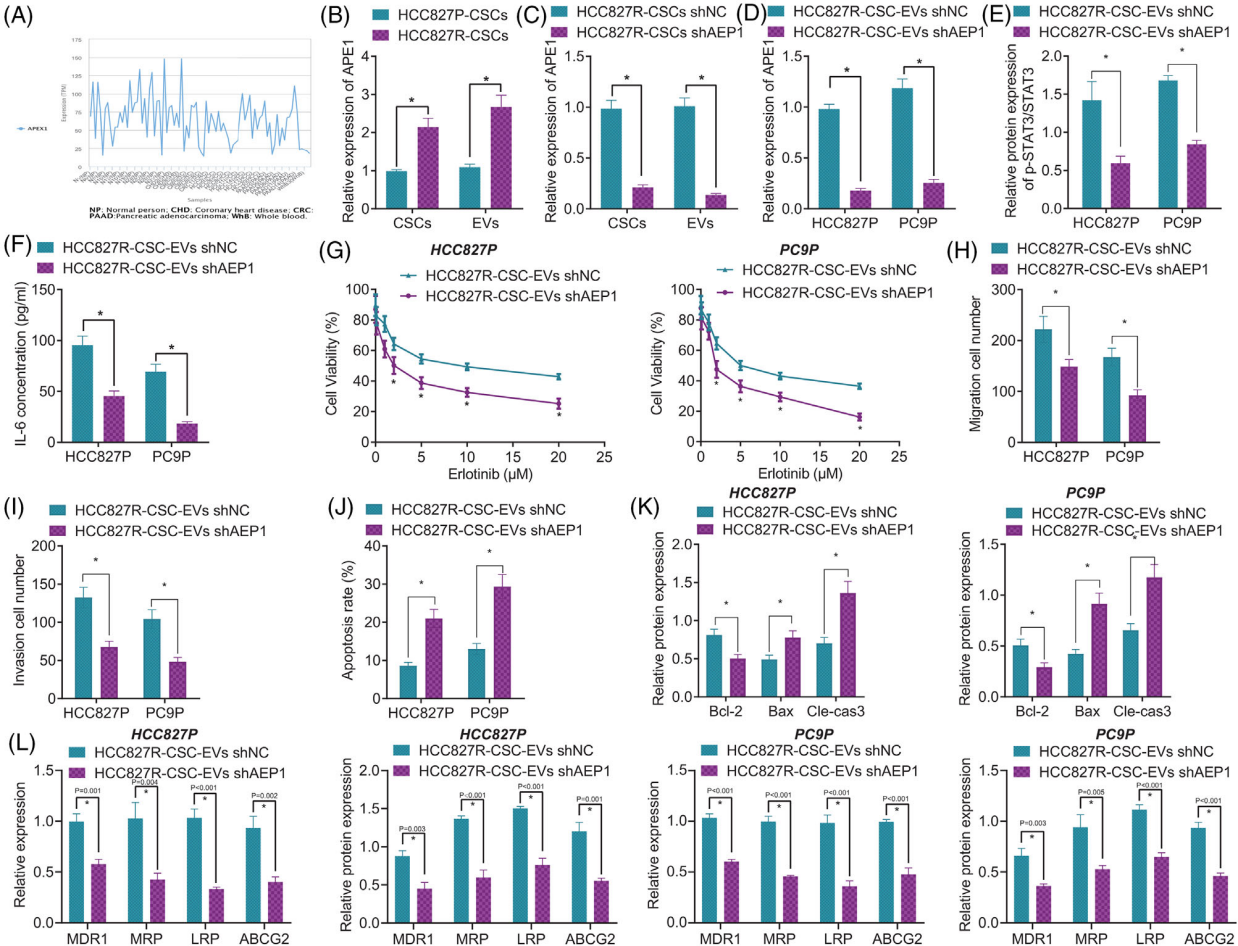
Effects of HCC827R‐CSC‐EVs loaded with APE1 shRNA on the Erlotinib resistance of NSCLC cells in vitro. (A) The enrichment of APEX1 (APE1) in EVs of different origins, analysed using the ExoRBase database. (B) The mRNA expression of APE1 in HCC827P‐CSCs, HCC827R‐CSCs, HCC827P‐CSC‐EVs and HCC827R‐CSC‐EVs determined by qRT‐PCR assay. (C) The mRNA expression of APE1 in HCC827R‐CSCs and HCC827R‐CSC‐EVs in response to shAPE1 treatment, determined by qRT‐PCR assay. (D) The mRNA expression of APE1 in HCC827P and PC9P cells in response to co‐culture with HCC827R‐CSCs EVs shAPE1 determined by qRT‐PCR assay. (E) Western blot measurement of the protein expression of p‐STAT3/STAT3 in HCC827P and PC9P cells in response to co‐culture with HCC827R‐CSCs EVs shAPE1. (F) ELISA detection of the content of IL‐6 in the supernatant of HCC827P and PC9P cells in response to co‐culture with HCC827R‐CSCs EVs shAPE1. (G) Cell viability in HCC827P and PC9P cells in response to co‐culture with HCC827R‐CSCs EVs shAPE1 and further treatment with Erlotinib, detected by CCK‐8 assay. Quantification of the migration (H) and invasion (I) in HCC827P and PC9P cells in response to co‐culture with HCC827R‐CSCs EVs shAPE1 and further treatment with Erlotinib (5 μM), detected by the Transwell assay. (J) Cell apoptosis in HCC827P and PC9P cells in response to co‐culture with HCC827R‐CSCs EVs shAPE1 and further treatment with Erlotinib (5 μM), detected by flow cytometry. (K) Protein expression of anti‐apoptotic Bcl‐2 and pro‐apoptotic Bax and cleaved caspase‐3 in the Western blot of Erlotinib (5 μM)‐treated HCC827P and PC9P cells in response to co‐culture with HCC827R‐CSCs EVs shAPE1. (L) The expression of MDR1, MRP, LRP and ABCG2 in Erlotinib (5 μM)‐treated HCC827P and PC9P cells in response to co‐culture with HCC827R‐CSCs EVs shAPE1 measured by qRT‐PCR and the Western blot. * *p *< .05. Each cell experiment was conducted in triplicate. APE1, apurinic endonuclease 1; CSC, cancer stem cell; EV, extracellular vesicle; IL‐6, interleukin‐6; NSCLC, non‐small cell lung cancer; shRNA, short hairpin RNA

As displayed by bioinformatics analysis using the ExoRBase database, APEX1 (APE1) was enriched in the EVs of various origins (Figure 5A). Then, APE1 was confirmed by qRT‐PCR analysis to be overexpressed in the RCSCs and corresponding RCSC‐EVs (all *p* < .01) (Figures 5B andS4A).

Further to explore whether CSC‐EVs delivered APE1 to affect the Erlotinib resistance of NSCLC cells, we first transduced RCSCs with lentivirus carrying shAPE1, isolated the EVs and co‐cultured them with HCC827P and PC9P cells. qRT‐PCR results identified that APE1 gene was successfully knocked down due to the fact that APE1 expression was significantly decreased (approximately 80%) in shAPE1‐treated RCSCs and RCSC‐EVs versus that in shNC‐treated RCSCs and RCSC‐EVs (all *p* < .01) (Figures 5C and S4B). In addition, the results of qRT‐PCR revealed that shAPE1‐treated HCC827P and PC9P cells, in response to co‐culture with RCSC‐EVs, presented with reduced APE1 expression (approximately 90%) versus the shNC‐treated cells co‐cultured with RCSC‐EVs (all *p* < .01) (Figures 5D and S4C). Western blot and ELISA data exhibited down‐regulated p‐STAT3/STAT3 and IL‐6 protein level upon treatment with RCSC‐EVs shAPE1 (*p*_HCC827P‐p‐STAT3/STAT3_ = .006; *p*_HCC827P‐IL‐6_ = .001; *p*_PC9P‐p‐STAT3/STAT3_ < .001; *p*_PC9P‐IL‐6_ < .001) (Figures 5E,F and S4D,E).

Moreover, HCC827P and PC9P cells co‐cultured with RCSC‐EVs were then further treated with different concentrations of Erlotinib. The results of the CCK‐8 assay showed a decreased viability of HCC827P and PC9P cells co‐cultured with RCSC‐EVs shAPE1 (*p*_HCC827P_ = .002; *p*_PC9P_ < .001) (Figures 5G and S4F). Next, 5‐μM Erlotinib was used to treat the HCC827P and PC9P cells co‐cultured with RCSC‐EVs. The results of the Transwell assay revealed that following the treatment with 5‐μM Erlotinib, HCC827P and PC9P cells co‐cultured with RCSC‐EVs shAPE1 showed attenuated migration (*p*_HCC827P_ = .012; *p*_PC9P_ = .003) and invasion (*p*_HCC827P_ = .002; *p*_PC9P_ = .002) (approximately 30%–60%) (Figures 5H,I, S3E,F, S4G,H). In addition, promoted apoptosis (approximately 50%–70%) was noted in HCC827P and PC9P cells co‐cultured with RCSC‐EVs shAPE1 following the treatment with 5‐μM Erlotinib (*p*_HCC827P_ = .001; *p*_PC9P_ = .001) (Figures 5J andS4I). Meanwhile, 5‐μM Erlotinib down‐regulated Bcl‐2 (*p*_HCC827P_ = .004; *p*_PC9P_ = .008) and up‐regulated Bax (*p*_HCC827P_ = .009; *p*_PC9P_ = .002) and cleaved caspase‐3 (*p*_HCC827P_ = .003; *p*_PC9P_ = .003) in HCC827P and PC9P cells co‐cultured with RCSC‐EVs shAPE1 relative to those co‐cultured with RCSC‐EVs shNC (Figures 5K andS4J).

Additionally, the results of qRT‐PCR and Western blot demonstrated a decline in the expression of MDR1, MRP, LRP and ABCG2 in HCC827P and PC9P cells co‐cultured with RCSC‐EVs shAPE1 in the presence of 5‐μM Erlotinib (Figures 5L and S4K).

Altogether, these results indicated that shAPE1‐loaded RCSC‐EVs could reduce the Erlotinib resistance of NSCLC cells by activating the IL‐6/STAT3 signalling.

### 3.6 shAPE1‐loaded HCC827R‐CSC‐EVs repress the Erlotinib resistance of NSCLC cells by activating the IL‐6/STAT3 signalling in vivo

**FIGURE 6:**
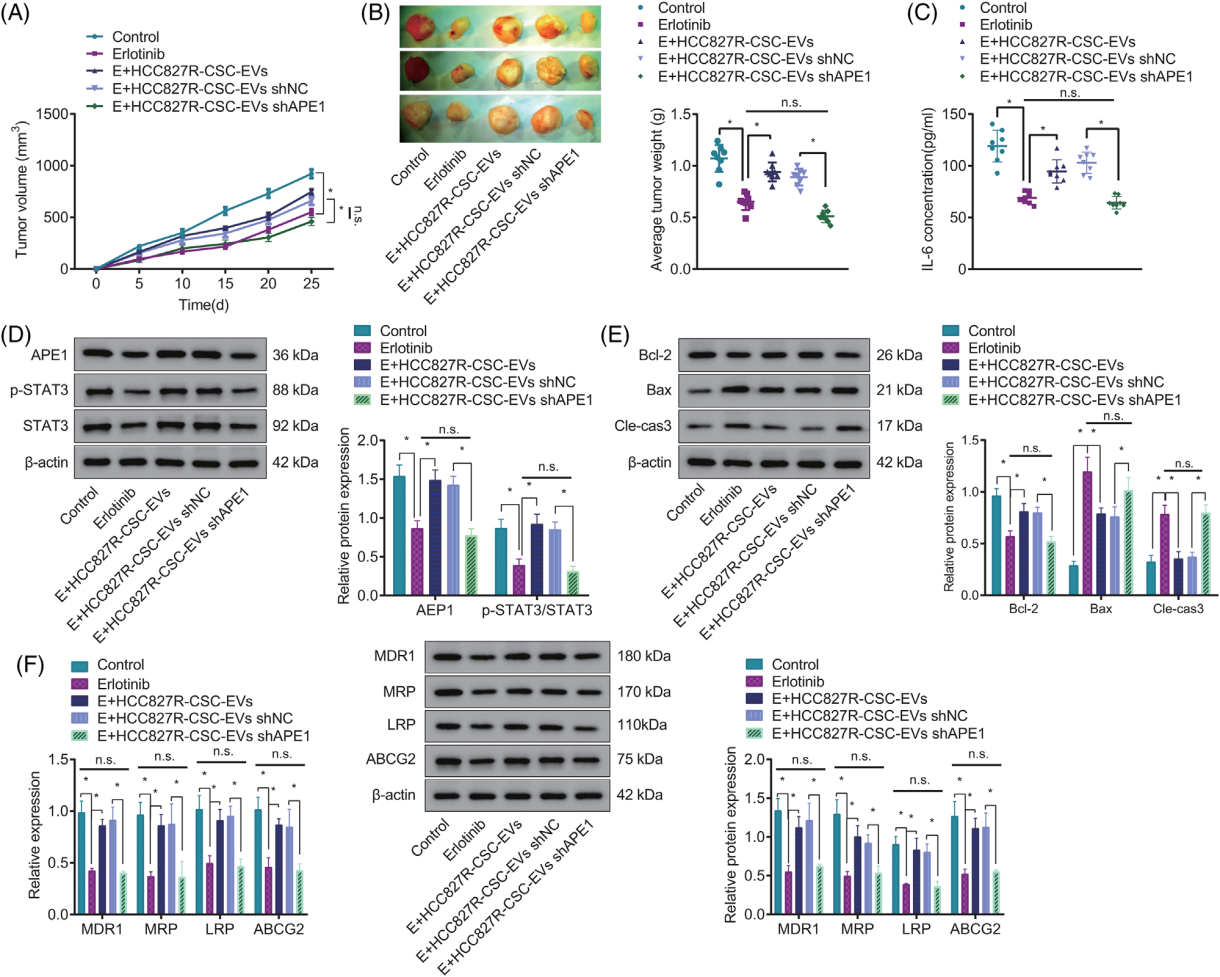
Effects of shAPE1‐loaded HCC827R‐CSC‐EVs on the Erlotinib resistance of NSCLC cells in nude mice. Nude mice were treated with Erlotinib, Erlotinib + HCC827R‐CSC‐EVs or Erlotinib + HCC827R‐CSC‐EVs shAPE1. (A) The volume of tumour xenografts at different time points. (B) The weight of isolated tumour xenografts in mice. (C) ELISA detection of IL‐6 protein levels in tumour tissues of mice. (D) The Western blot measurement of the protein expression of APE1, p‐STAT3 and STAT3 in tumour tissues of mice. (E) The Western blot measurement of anti‐apoptotic Bcl‐2 and pro‐apoptotic Bax and cleaved caspase‐3 in tumour tissue of mice. (F) The expression of MDR1, MRP, LRP and ABCG2 in tumour tissues of mice measured by qRT‐PCR and Western blot. * *p* < .05. *n* = 8. APE1, apurinic endonuclease 1; CSC, cancer stem cell; EV, extracellular vesicle; IL‐6, interleukin‐6; NSCLC, non‐small cell lung cancer

To further verify the effect of RCSC‐EVs‐APE1 on the Erlotinib resistance of NSCLC in vivo, we constructed a xenograft model of NSCLC in nude mice by a subcutaneous injection of HCC827P cells and then used injected the mice with the HCC827R‐CSC‐EVs isolated from APE1 shRNA lentivirus‐transduced HCC827R‐CSCs or Erlotinib. Next, the volume and weight of the xenograft tumour were observed and recorded. It was observed in NSCLC mouse models that the tumour volume and weight were attenuated (approximately 50%–60%; all *p* < .01) in response to the Erlotinib treatment than without any treatment, and the inhibiting effects of Erlotinib were reversed by HCC827R‐CSC‐EVs (approximately 50%; all *p* < .01); versus mice treated with Erlotinib + HCC827R‐CSC‐EVs shNC, those treated with Erlotinib + HCC827R‐CSC‐EVs shAPE1 presented with reduced tumour volume and weight (approximately 50%; all *p* < .01) (Figure 6A,B).

Further, the results of Western blot and ELISA demonstrated that the Erlotinib treatment led to increased levels of APE1, IL‐6 and p‐STAT3/STAT3 in tumour tissues, which could further be increased in response to HCC827R‐CSC‐EVs (all *p* < .01). Relative to HCC827R‐CSC‐EVs shNC, HCC827R‐CSC‐EVs shAPE1 resulted in down‐regulated levels of APE1, IL‐6 and p‐STAT3/STAT3 in tumour tissues of Erlotinib‐treated mice (all *p* < .01) (Figure 6C,D).

Moreover, decreased Bcl‐2 and increased Bax and cleaved caspase‐3 were noted in tumour tissues from mice treated with Erlotinib, and these effects of Erlotinib were abrogated by the injection of HCC827R‐CSC‐EVs (all *p* < .01). Compared to Erlotinib + HCC827R‐CSC‐EVs shNC, Erlotinib + HCC827R‐CSC‐EVs shAPE1 led to down‐regulated level of Bcl‐2 as well as up‐regulated levels of Bax and cleaved caspase‐3 (all *p* < .01) (Figure 6E).

Moreover, the results of qRT‐PCR and Western blot demonstrated a decline in the expression of MDR1, MRP, LRP and ABCG2 in tumour tissues of mice treated with Erlotinib, the effect of which was abolished by HCC827R‐CSC‐EVs. However, in the presence of Erlotinib + HCC827R‐CSC‐EVs shAPE1, the expression of MDR1, MRP, LRP and ABCG2 was decreased compared to Erlotinib + HCC827R‐CSC‐EVs shNC (Figure 6F).

In summary, shAPE1‐loaded HCC827R‐CSC‐EVs could reduce the resistance of NSCLC cells to Erlotinib in vivo by activating the IL‐6/STAT3 signalling.

## 4 DISCUSSION

**FIGURE 7:**
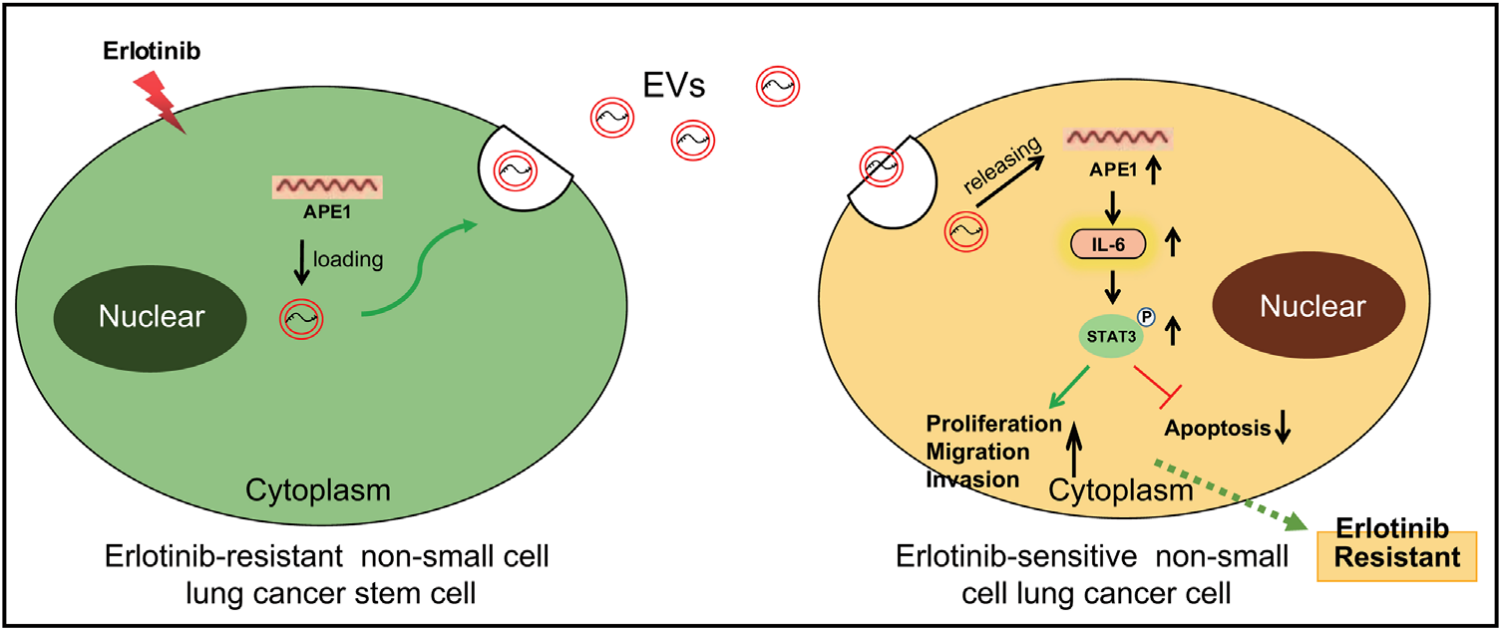
The molecular mechanism underlying the effect of shAPE1‐loaded RCSC‐EVs on the Erlotinib resistance in NSCLC. shAPE1‐loaded RCSC‐EVs suppress the activation of the IL‐6/STAT3 signalling and thereby reverse the resistance of NSCLC cells to Erlotinib. EV, extracellular vesicle; IL‐6, interleukin‐6; NSCLC, non‐small cell lung cancer

Erlotinib is a TKI capable of inhibiting EGFR, and patients with NSCLC show tumour response, survival and tumour‐related symptom improvement,^22^, ^23^ whereas its efficacy is limited by inevitable drug resistance developed in long‐term treatment.^10^, ^24^ The present study defined a pivotal role of EVs derived from Erlotinib‐resistant CSCs in the Erlotinib resistance of NSCLC and that RCSC‐EVs delivered APE1 into NSCLC to induce NSCLC resistance to Erlotinib by activating the IL‐6/STAT3 signalling (Figure 7).

Increasing evidence supports that tumour growth is fuelled by CSCs, a highly tumorigenic subpopulation, and that CSCs are responsible for the metastasis and therapeutic resistance of cancer^25^, ^26^, ^27^ as these cells largely contribute to the stem‐like phenotypes in cancer cells.^28^ In our study, we found that in addition to its own possible drug resistance, CSCs can also promote the transformation of sensitive NSCLC cells into drug‐resistant cells by secreting EVs. Initially, we demonstrated that although EVs isolated from either parental or Erlotinib‐resistant CSCs (PCSC‐EVs, RCSC‐EVs) could be internalized by NSCLC cells, only EVs isolated from Erlotinib‐resistant CSCs can significantly promote the Erlotinib resistance of tumour cells. A previous study indicated that glioma stem cells secrete the pro‐angiogenic VEGF‐A factor through EVs, and the EV‐loaded VEGF‐A may affect the formation of brain endothelial cells into new vessels.^29^ In pancreatic cancer where chemotherapy by gemcitabine (GEM) is a first‐line treatment for locally advanced cases, EVs released by GEM‐resistant pancreatic CSCs have been suggested to modulate drug‐resistant trait horizontal transfer to GEM‐sensitive pancreatic cancer cells through shuttling microRNA‐210 (miR‐210).^12^ Therefore, we do not rule out that drug‐resistant tumour NSCLC cells can exclude Erlotinib drugs from the body through EVs, so as to realize their resistance to Erlotinib. However, from our results, EVs mostly transmit some drug resistance factors and promote the drug resistance of surrounding sensitive cells.

Further to explore the underlying mechanisms, we unveiled through microarray profiling that IL‐6 expression was up‐regulated in Erlotinib‐resistant NSCLC samples. Intriguingly, Clay et al. have indicated that the up‐regulation of IL‐6/STAT signal transduction may be a contributor to EGFR‐TKIs resistance in lung cancer.^13^ The transduction of the IL‐6 signal via binding to glycoprotein 130 has been established to induce the activation of Janus kinase, which could then lead to the activation of STAT^30^; and the aberrant hyperactivation of the IL‐6/STAT signalling has been highlighted in various cancer types, correlated with a poor clinical prognosis.^31^ Furtherly, polyphyllin I overcomes the Erlotinib resistance in NSCLC cells via IL‐6/STAT3 signalling inhibition.^14^ Our results are highly consistent with these findings. The activation of IL‐6/STAT3 signalling does exert a critical role in the Erlotinib resistance of NSCLC.

At present, many studies have highlighted the critical role of IL‐6/STAT3 signalling in the Erlotinib resistance of NSCLC. However, so far, few articles have reported upstream regulators of IL‐6/STAT3 in the regulation of Erlotinib resistance in NSCLC. On the basis of the aforementioned reports, our data displayed the up‐regulated expression of APE1 in NSCLC tissues as well as an elevated IL‐6 level in the serum of patients with lung cancer. The extracellular role of DNA damage repair protein APE1 has been suggested by prior studies, wherein extracellular APE1 was reported to trigger the IL‐6 signalling and thereby augment IL‐6‐induced autocrine and paracrine actions.^15^, ^32^ APE1 overexpression significantly contributes to the TKI resistance via activating the Akt signalling via a redox‐dependent mechanism in LUAD cells.^33^ Moreover, Liu et al. revealed in their investigations that mitochondrial APE1 may promote the resistance to Cisplatin, a well‐known cancer chemotherapeutic agent, by down‐regulating reactive oxygen species in osteosarcoma.^34^, ^35^ Suppression of APE1 expression has also been recognized to be a promising method to prevent bortezomib resistance in multiple myeloma, a malignant neoplasm of plasma cells accumulated in the bone marrow.^36^ Collectively, we assumed that the APE1/IL‐6/STAT3 signalling participated in the modulation of Erlotinib resistance in NSCLCs, and we demonstrated shAPE1‐loaded RCSC‐EVs could reduce the resistance of NSCLC to Erlotinib.

However, previous reports have found that IL‐6/STAT3 is regulated by many different regulatory factors,^37^, ^38^, ^39^ so whether there are other factors involved in the Erlotinib resistance regulation needs to be further screened and explored in the future.

## 5 CONCLUSIONS

In conclusion, data acquired in the present study illuminated that APE1‐loaded RCSC‐EVs can promote Erlotinib resistance in NSCLC by promoting the activation of the IL‐6/STAT3 signalling (Figure 7). Therefore, these data provided an insight into the pathogenesis of the Erlotinib resistance in NSCLC, which may shed light on novel approaches to overcome the Erlotinib resistance in the management of NSCLC. Nonetheless, the relationship between APE1 expression and the Erlotinib resistance of patients with NSCLC is required based on enough samples of Erlotinib‐resistant and sensitive NSCLC patients.

## CONFLICT OF INTEREST

The authors declare that they have no competing interests.

## Supporting information

Supporting Information
