# Supplementary material for: APE1 shRNA‐loaded cancer stem cell‐derived extracellular vesicles reverse Erlotinib resistance in non‐small cell lung cancer via the IL‐6/STAT3 signalling
Source: Clin Transl Med. 2022 May 23;12(5):e876. doi: 10.1002/ctm2.876 (PMC9126360; doi:10.1002/ctm2.876)
Supplement: Supplementary file 1 — Supporting Information [file CTM2-12-e876-s001.docx]

**Supporting Information**

**
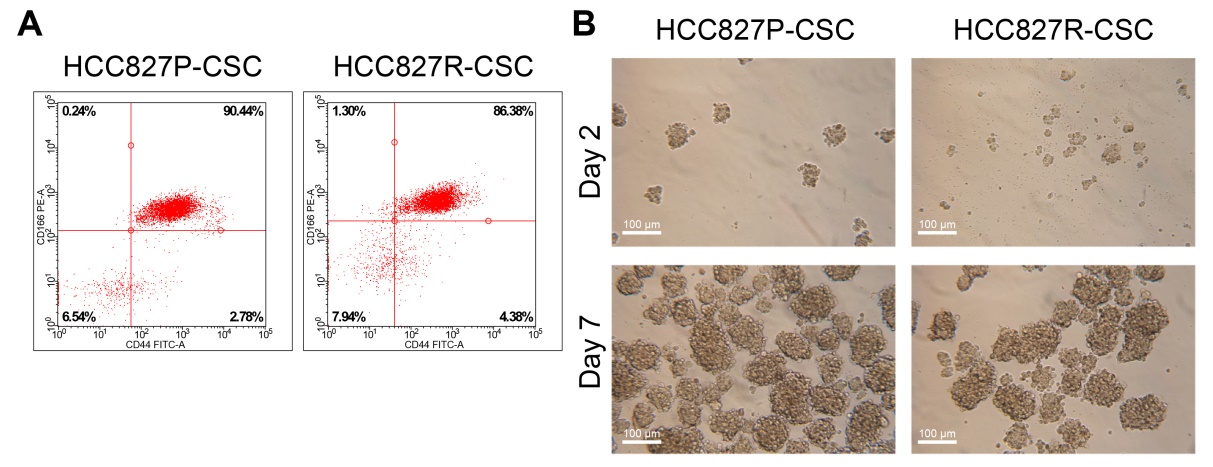
**

**Figure S1** Successful isolation of HCC827P-CSCs and HCC827R-CSCs. A, the expression of CD44 and CD166 in the isolated CD133^+^ABCG2^+^ HCC827P-CSCs and HCC827R-CSCs determined by flow cytometry. B, Sphere-forming ability of the isolated HCC827P-CSCs and HCC827R-CSCs determined by sphere-forming experiment.

**
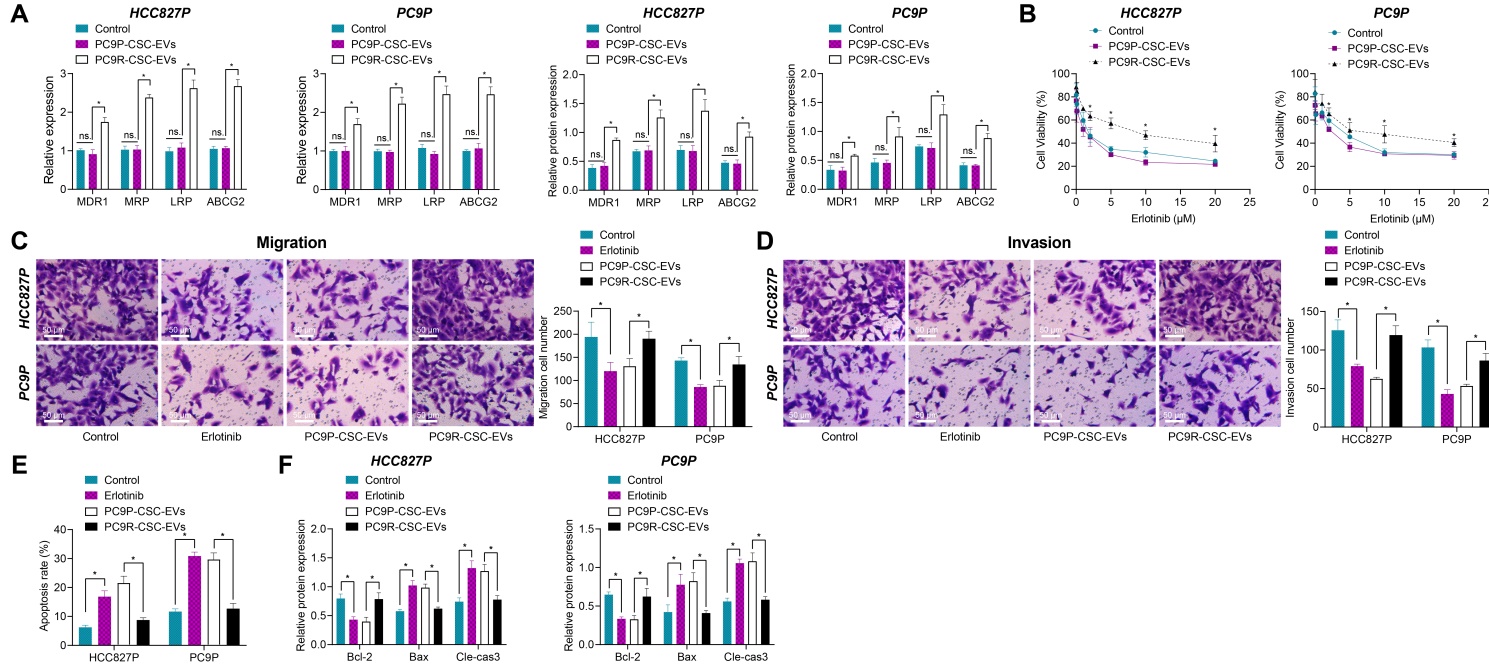
**

**Figure S2** Effect of PC9P-CSC-EVs and PC9R-CSC-EVs on the resistance of NSCLC cells to Erlotinib. A, Expression of Erlotinib resistance-related genes MDR1, MRP, LRP and ABCG2 in HCC827P and PC9P cells following co-culture with PC9P-CSC-EVs or PC9R-CSC-EVs, detected by qRT-PCR and Western blot. B, Viability of HCC827P and PC9P cells co-cultured with PC9P-CSC-EVs or PC9R-CSC-EVs and further treated with different concentrations of Erlotinib, detected by CCK-8 assay. C-D, Representative images and corresponding quantification of the migration (C) and invasion (D) of Erlotinib (5 μM)-treated HCC827P and PC9P cells in response to co-culture with PC9P-CSC-EVs or PC9R-CSC-EVs, observed by Transwell assay. E, Apoptosis of Erlotinib (5 μM)-treated HCC827P and PC9P cells in response to co-culture with PC9P-CSC-EVs or PC9R-CSC-EVs, detected by flow cytometry. F, Protein expression of anti-apoptotic Bcl-2 and pro-apoptotic Bax and cleaved caspase-3 in Western blot of Erlotinib (5 μM)-treated HCC827P and PC9P cells in response to co-culture with PC9P-CSC-EVs or PC9R-CSC-EVs. * *p* < 0.05, Each cell experiment was conducted in triplicate.

**
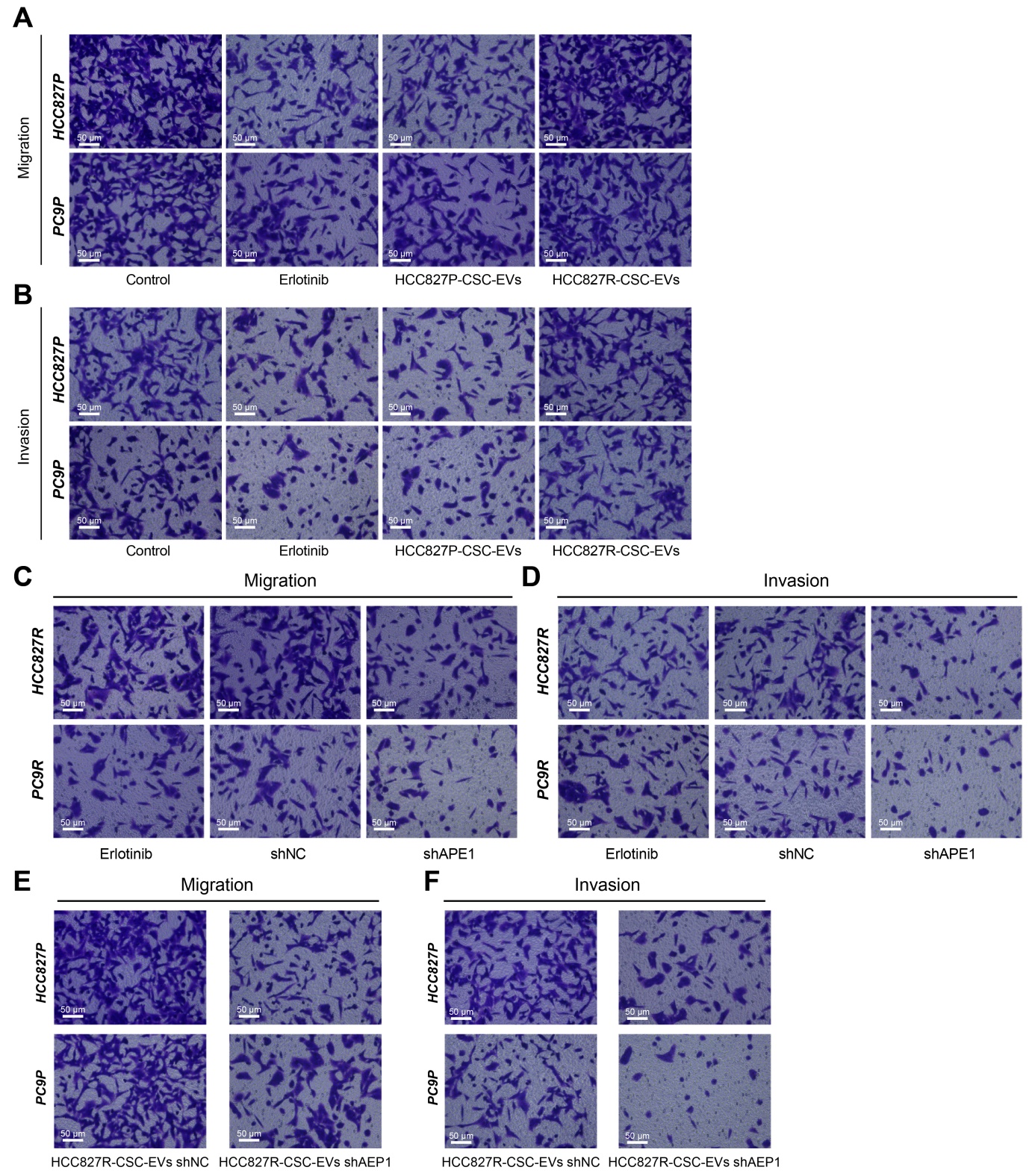
**

**Figure S3** Representative images of the migration and invasion. A-B, Representative images of the migration (A) and invasion (B) of Erlotinib (5 μM)-treated HCC827P and PC9P cells in response to co-culture with HCC827P-CSC-EVs or HCC827R-CSC-EVs, observed by Transwell assay. C-D, Representative images of the migration (C) and invasion (D) in HCC827R and PC9R cells treated by Erlotinib alone or in combination with shAPE1, observed in Transwell assay. E-F, Representative images of the migration (E) and invasion (F) in HCC827P and PC9P cells in response to co-culture with HCC827R-CSCs EVs shAPE1 and further treatment with Erlotinib (5 μM), detected by Transwell assay.

**
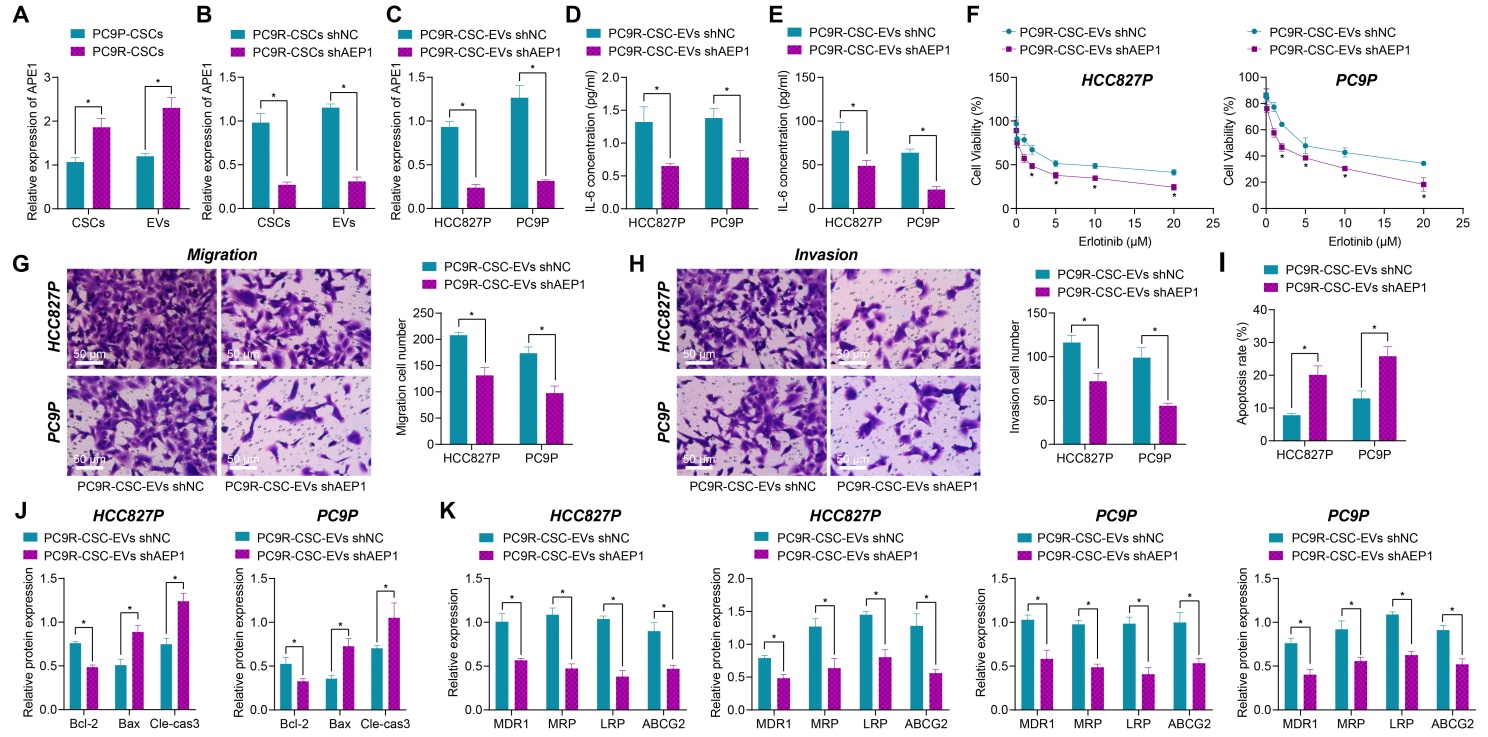
**

**Figure S4** Effects of PC9R-CSC-EVs loaded with APE1 shRNA on the Erlotinib resistance of NSCLC cells in vitro. A, mRNA expression of APE1 in PC9P-CSCs, PC9R-CSCs, PC9P-CSC-EVs and PC9R-CSC-EVs determined by qRT-PCR assay. B, The mRNA expression of APE1 in PC9R-CSCs and PC9R-CSC-EVs in response to shAPE1 treatment, determined by qRT-PCR assay. C, The mRNA expression of APE1 in HCC827P and PC9P cells co-cultured with PC9R-CSC-EVs shAPE1, determined by qRT-PCR assay. D, Western blot measurement of the protein expression of p-STAT3/STAT3 in HCC827P and PC9P cells in response to co-culture with PC9R-CSC-EVs shAPE1. E, ELISA detection of the content of IL-6 in the supernatant of HCC827P and PC9P cells in response to co-culture with PC9R-CSC-EVs shAPE1. F, Cell viability in HCC827P and PC9P cells in response to co-culture with PC9R-CSC-EVs shAPE1 and further treatment with Erlotinib. G, H, Representative images and corresponding quantification of the migration (H) and invasion (I) in HCC827P and PC9P cells in response to the co-culture with PC9R-CSC-EVs shAPE1 and further treatment with Erlotinib (5 μM), detected by Transwell assay. I, Cell apoptosis in HCC827P and PC9P cells in response to co-culture with PC9R-CSC-EVs shAPE1 and further treatment with Erlotinib (5 μM), detected by flow cytometry. J, Protein expression of anti-apoptotic Bcl-2 and pro-apoptotic Bax and cleaved caspase-3 in Western blot of Erlotinib (5 μM)-treated HCC827P and PC9P cells in response to co-culture with PC9R-CSC-EVs shAPE1. K, The expression of MDR1, MRP, LRP and ABCG2 in Erlotinib (5 μM)-treated HCC827P and PC9P cells in response to co-culture with PC9R-CSC-EVs shAPE1 measured by qRT-PCR and Western blot. * *p* < 0.05. Each cell experiment was conducted in triplicate.

**Supplementary Table 1** Primer sequences for qRT-PCR

| Genes | Sequence (5' - 3') |
| --- | --- |
| APE1 (human) | Forward: TGTGTGGAGACCTCAATGTG |
|  | Reverse: GTAGGCATAGGGTGTGTTGG |
| MDR1 (human) | Forward: CCCATCATTGCAATAGCAGG |
|  | Reverse: GTTCAAACTTCTGCTCCTGA |
| MRP (human) | Forward: GTCACGTGGAATACCAGCAAC |
|  | Reverse: GTCCACAGACATGAGGTTGAC |
| LRP (human) | Forward: AGCTACCTGCACTTCTAGATTCA |
|  | Reverse: ATGTAGGTCTTTGGCCCGAC |
| ABCG2 (human) | Forward: GGAACTCAGTTTATCCGTGG |
|  | Reverse: CGAGGCTGATGAATGGAGAAG |
| β-actin (human) | Forward: GATCATTGCTCCTCCTGAGC |
|  | Reverse: TGTGGACTTGGGA GAGGACT |
